# New function of the myostatin/activin type I receptor (ALK4) as a mediator of muscle atrophy and muscle regeneration

**DOI:** 10.1096/fj.201600675R

**Published:** 2016-10-12

**Authors:** Svitlana Pasteuning-Vuhman, Johanna W. Boertje-van der Meulen, Maaike van Putten, Maurice Overzier, Peter ten Dijke, Szymon M. Kiełbasa, Wibowo Arindrarto, Ron Wolterbeek, Ksenia V. Lezhnina, Ivan V. Ozerov, Aleksandr M. Aliper, Willem M. Hoogaars, Annemieke Aartsma-Rus, Cindy J. M. Loomans

**Affiliations:** * Department of Human Genetics, Leiden University Medical Center, Leiden, The Netherlands;; † Department of Molecular and Cell Biology Leiden University Medical Center, Leiden, The Netherlands;; ‡ Cancer Genomics Center, Leiden University Medical Center, Leiden, The Netherlands;; § Department of Medical Statistics and Bioinformatics, Leiden University Medical Center, Leiden, The Netherlands;; ¶ InSilico Medicine, Incorporated, Emerging Technology Centers, Johns Hopkins University, Baltimore, Maryland, USA; and; ‖ Department of Human Movement Sciences, Faculty of Behavioral and Movement Sciences, Move Research Institute Amsterdam, Vrije Universiteit (VU) Amsterdam, Amsterdam, The Netherlands

**Keywords:** Duchenne muscular dystrophy, antisense oligonucleotides, myostatin/activin pathway, muscle metabolism, muscle mass

## Abstract

Skeletal muscle fibrosis and impaired muscle regeneration are major contributors to muscle wasting in Duchenne muscular dystrophy (DMD). Muscle growth is negatively regulated by myostatin (MSTN) and activins. Blockage of these pathways may improve muscle quality and function in DMD. Antisense oligonucleotides (AONs) were designed specifically to block the function of ALK4, a key receptor for the MSTN/activin pathway in skeletal muscle. AON-induced exon skipping resulted in specific *Alk4* down-regulation, inhibition of MSTN activity, and increased myoblast differentiation *in vitro*. Unexpectedly, a marked decrease in muscle mass (10%) was found after *Alk4* AON treatment in *mdx* mice. In line with *in vitro* results, muscle regeneration was stimulated, and muscle fiber size decreased markedly. Notably, when *Alk4* was down-regulated in adult wild-type mice, muscle mass decreased even more. RNAseq analysis revealed dysregulated metabolic functions and signs of muscle atrophy. We conclude that ALK4 inhibition increases myogenesis but also regulates the tight balance of protein synthesis and degradation. Therefore, caution must be used when developing therapies that interfere with MSTN/activin pathways.—Pasteuning-Vuhman, S., Boertje-van der Meulen, J. W., van Putten, M., Overzier, M., ten Dijke, P., Kiełbasa, S. M., Arindrarto, W., Wolterbeek, R., Lezhnina, K. V., Ozerov, I. V., Aliper, A. M., Hoogaars, W. M., Aartsma-Rus, A., Loomans, C. J. M. New function of the myostatin/activin type I receptor (ALK4) as a mediator of muscle atrophy and muscle regeneration.

Myostatin (MSTN) is a member of the TGF-β family and an inhibitor of muscle growth. It is expressed in skeletal muscle and, to a lesser extent, in adipose tissue and cardiac muscle (1–3). MSTN mainly acts *via* interaction with the type II receptor ACVR2B, which forms a heterodimer with the type I receptors ACVR1B (ALK4) and TGFBR1 (ALK5) (4, 5). The intracellular serine/threonine domains of ALK4 and -5 phosphorylate Smad2 and -3 proteins, which form a complex with Smad4 (6). This complex enters the nucleus and regulates the transcription of target genes, including genes involved in muscle growth, muscle metabolism, and fibrosis (7, 8). Lack of MSTN caused by spontaneous mutations or genetic knockout in mammals (including humans) causes skeletal muscle hyperplasia and hypertrophy. MSTN-knockout mice show improved muscle regeneration upon muscle damage (8, 9). Inhibition of MSTN is considered as a promising therapy for muscle-wasting disorders, including Duchenne muscular dystrophy (DMD), a lethal and common form of muscular dystrophy affecting ∼1 in 5000 newborn boys worldwide (10, 11). Patients are wheelchair bound from ∼12 yr, need assisted ventilation at ∼20 yr, and usually die in the third or fourth decade. Several *in vivo* studies in *mdx* mice (DMD mouse model) showed that MSTN inhibition was well tolerated and beneficial, with increased muscle mass and improved function (9, 12–14). In the past few years, blockage of the MSTN/ACVR2B pathway as a therapeutic strategy for muscular dystrophies, muscle wasting, and cachexia has been investigated in multiple clinical trials (NCT01099761, NCT01519349, NCT01423110, NCT01669174, NCT01601600, and NCT01433263) [National Institutes of Health (NIH), Bethesda, MD, USA; *https://clinicaltrials.gov*]. In a clinical trial in adult patients with muscular dystrophy, MSTN-neutralizing antibodies were well tolerated but had no beneficial effects on the muscles (15). Another strategy, systemic administration of a soluble form of the type II receptor ACVR2B ligand binding domain (ActRIIB-Fc), was well tolerated by healthy postmenopausal women and achieved a 5% increase in lean body mass at the highest doses (16). A subsequent phase II trial in patients with DMD was terminated because of adverse events (nose/gum bleeding) (NCT01099761), ascribed to interference of the soluble receptor with activin signaling.

We have shown that MSTN signaling occurs *via* the ALK4 receptor in myogenic cells and *via* ALK5 in nonmyogenic cells, including muscle fibroblasts (17). We also reported an antisense oligonucleotide (AON)–mediated splicing modulation strategy that could interfere with the TGF-β signaling pathways (18). Interference was achieved by knockdown of *Alk5* expression with AONs that target the in-frame exon 2 of ALK5. Treatment with these AONs decreases fibrotic gene expression and increases myogenic gene expression in *mdx* mice. In the present study, we selectively targeted ALK4, to block MSTN/activin signaling, aiming at increasing the muscle mass in *mdx* mice. Unexpectedly, we found that this triggered loss in muscle mass and an increase in muscle regeneration in the *mdx* mice. In adult wild-type (WT) mice, *Alk4* down-regulation resulted in an even more pronounced loss of muscle mass. To better understand the underlying mechanism, RNA sequencing (RNA-seq) analysis was performed on AON-treated WT muscles. Based on this analysis, we suggest that ALK4 signaling is a key mediator of muscle growth and wasting.

## MATERIALS AND METHODS

### Ethics statements

All experiments were approved by and performed according to the guidelines of the Animal Experiment Committee (Dierexperimentencommissie) of the Leiden University Medical Center. Care was taken to limit the burden and distress for the animals as much as possible.

### Cell cultures and AON transfections

Mouse myoblasts C2C12 [American Type Culture Collection (ATCC, Manassas, VA, USA)] were maintained in proliferation medium containing DMEM with 10% fetal bovine serum (FBS), 1% glucose, and 2% Glutamax (Thermo Fisher Scientific, Waltham, MA, USA) at 37°C with 5% CO_2_. Mesenchymal stem cells C3H10 T1/2 (ATCC) were grown in α-MEM with 10% FBS at 37°C with 5% CO_2_. The differentiation medium for C2C12 was DMEM with 2% FBS, 1% glucose, and 2% Glutamax. Primary myoblasts were isolated from extensor digitorum longus (EDL) muscles of 2-mo-old *mdx* mice and digested in collagen type 1 as previously described (19, 20). Single myofibers were cultured on Matrigel (Corning, Nieuwegein, The Netherlands) for 3 d in serum-containing (SC) medium, composed of DMEM supplemented with 30% FBS, 10% horse serum, 1% glucose, 2% Glutamax, 1% chicken embryonic extract (Bio-Connect, Huissen, The Netherlands), 10 ng/ml basic fibroblast growth factor (Promega, Leiden, The Netherlands), and 1% penicillin–streptomycin (Thermo Fisher Scientific) at 37°C with 5% CO_2_, allowing migration of satellite cells from isolated myofibers cultured on Matrigel. A preplating step was performed to remove muscle fibroblasts: cells were transferred to noncoated culturing flasks and incubated for 20 min. Nonattached satellite cells were then replated on Matrigel at equal density and maintained in SC medium. Differentiation medium for primary myoblasts consisted of DMEM with 2% horse serum, 1% glucose, and 2% Glutamax. AONs with full-length phosphorothioate backbones and 2′-*O*-methyl-modified RNA were kindly provided by BioMarin Nederland BV (Leiden, The Netherlands). The sequences are listed in Table 1. The control AON was designed as a scrambled sequence of *Alk4* AON. For transfection, we used Lipofectamine 2000 (Thermo Fisher Scientific), according to the manufacturer’s instructions. In short, C2C12 myoblasts were transfected with a mixture of 100 nM AON and Lipofectamine. Separate AON–Lipofectamine dilutions were made for each AON with 3.5 μl Lipofectamine applied per 100,000 cells per 1 ml. For primary myoblasts, we used 50 nM of each AON Lipofectamine and 2.5 μl of Lipofectamine per 100,000 cells per 1 ml. Transfection was performed in penicillin-streptomycin–free differentiation medium and 4 h later, the medium was refreshed. C2C12 myoblasts were also transfected with vivo-morpholinos (ViMs) targeting *Alk4* transcript at the final concentration of 10, 5, 2, and 1 μM, without any transfection reagents in the differentiation medium, and incubated for 72 h.

**TABLE 1.** Sequences of 2OMePS AONs and ViMs

Name	Chemistry	Target gene/exon	Sequence, 5′–3′
*Alk4* AON	2OMePS	*Alk4*/exon6	UGACUUCAAGUCUCGAUGAGC
Scr*Alk4* AON	2OMePS	Nontargeting	CUAGGUCAAUCGAUGCUUGAC
*Alk4* ViM	ViM	*Alk4*/exon6	TCTATGGTGTCAGTGACCGCATCAT
Control ViM	ViM	Nontargeting	CCTCTTACCTCAGTTACAATTTATA

### Myogenic differentiation assay

C2C12 cells were plated on collagen-coated 6-well plates (for myogenic gene expression analysis) and 12-well plates (for immunofluorescence staining) at a density of 100,000 cells/ml proliferation medium. After 24 h, the cells were transfected with *Alk4* or control AON with Lipofectamine. Four hours after transfection, differentiation was induced by culturing in low serum. At differentiation d 3, the cells were transfected a second time with *Alk4* or scrambled *Alk4* AONs. The progression of differentiation was monitored for 5 d, after which the expression of myogenic genes was analyzed. The differentiation index was calculated as the percentage of the total number of myogenic cells that were myosin positive. Primary myoblasts isolated from *mdx* EDL muscle were plated on Matrigel-coated 6-well plates (for myogenic gene expression analysis) at a density of 100,000 cells/ml SC medium. After 24 h, the cells were transfected with ALK4 or scrambled *Alk4* AON using Lipofectamine. Four hours after transfection, myogenic differentiation was initiated by culturing in differentiation medium for 2–3 d.

### Luciferase reporter assay

C2C12 or C3H10 T1/2 cells were seeded at a density of 5000 cells/well on white clear bottom 96-well plates (Greiner Bio-One, Alphen aan den Rijn, The Netherlands) until 70% confluence and transiently transfected with 100 ng (CAGA)_12_-Luc, 10 ng pRL-CMV, and 200 nM of the indicated AON with Dharmafect Duo (ThermoFisher Scientific Life Sciences). After an overnight serum starvation, cells were preincubated for 1 h with 10 μM LY364947 (ALK4/5/7 kinase inhibitor; Sigma-Aldrich, Zwijndrecht, The Netherlands) as a positive control for blockage of MSTN signaling. After the preincubation, cells were stimulated with 500 ng/ml MSTN (R&D Systems, Abingdon, United Kingdom) and/or 10 μM LY364947 or serum-free medium for 8 h. The cells were lysed with the DualGlo Luciferase Assay Kit (Promega, Leiden, The Netherlands), and luciferase signals were read in the Multilabel Counter (Perkin Elmer, Waltham, MA, USA). *Renilla* luciferase signals served to normalize for transfection efficiency.

### ViM treatment in mice

For animal experiments, ViM counterparts of the most efficient *Alk4* AON were purchased from Gene-Tools (Philomath, OR, USA). Sequences are listed in Table 1. Mice were bred by the animal facility of the Leiden University Medical Center and kept in ventilated caged with 12 h of light dark cycles. Mice were age and gender matched and were randomized over the experimental groups. Investigators performing the analyses were blinded to experimental group. Gastrocnemius and tibialis anterior muscles of 4-wk-old male *mdx* or 3-mo-old male C57BL/6Jico WT mice were injected with 40 μg ALK4 ViM or control nontargeting ViM at the same dose. Mice were anesthetized with 2% isoflurane, intramuscularly injected on 2 consecutive days, and euthanized 1 wk (short-term treatment) or 7 wk (long-term treatment) after the first injection. Long-term treatment included an extra injection 4 wk after the first injection. Gastrocnemius and tibialis anterior muscles were isolated and snap frozen in 2-methylbutane (Sigma-Aldrich) cooled in liquid nitrogen. Sections of 8 µm were obtained with a microtome used for RNA isolation and histologic analyses.

### *In vivo* force measurements of tibialis anterior muscles

The same long-term, 3-injection protocol was used for the tibialis anterior muscles as for the gastrocnemius muscles. Each mouse was anesthetized with 2% isoflurane 7 wk after the first intramuscular *Alk4* ViM or control ViM injection (21). In short, the tendon of the tibialis anterior was exposed and freed, after which a thread was tightly secured and tied into a loop. The sciatic nerve was located, and all branches of the nerve were cut while keeping the common peroneal nerve, which stimulates the tibialis anterior, intact. The mouse was placed on a rig, and the leg was immobilized by a needle stuck between the patella and the knee and secured in the rig setup, while the foot was secured with tape. In this way, any unwanted movements of the leg were prevented. The tendon was attached *via* a homemade S-hook to the lever arm of the force transducer, and the distal part of the peroneal nerve was stimulated with bipolar platinum electrodes. Data were acquired with a Lab-View-based DMC program (Dynamic Muscle Control and Data Acquisition; Aurora Scientific, Aurora, ON, Canada). After an initial warmup (5 stimulations of 50 Hz spaced 1 min apart), optimal muscle length (*L*_o_) was determined by a series of twitches at increasing resting tension. Maximum isometric tetanic force (*P*_o_) was determined from the plateau of the force–frequency relationship after a series of stimulations at 10, 30, 40, 50, 80, 100, 120, 150, and 180 Hz, spaced 1 min apart. The specific force (N/cm^2^) was determined by dividing *P*_o_ by muscle cross-sectional area. Overall cross-sectional area was estimated with the following formula: muscle weight (g)/[TA fiber length (*L*_f_; cm) × 1.06 g/cm^3^]. Specific isometric force was determined by dividing the absolute force at each stimulation frequency by the muscle’s cross-sectional area. After a resting period of 5 min, the susceptibility of tibialis anterior muscles to contraction-induced injury was measured (21). The muscles were stimulated at 120 Hz for 500 ms before lengthening at 10% of *L*_o_ at a velocity of 0.5 *L*_o_ s^−1^. At the end of the stimulation, the muscle was returned to *L*_o_ at a rate of −0.5 *L*_o_ s^−1^. The stimulation–stretch cycle was repeated every 2 min for a total of 10 cycles. The maximum isometric force of the second contraction was used as 100% baseline. After the procedure, the muscles were isolated, weighed, and prepared for RNA analysis.

### RNA isolation, RT-PCR, and quantitative PCR

Muscle tissues were sectioned with a cryotome, and the sections were collected in 1.4-mm zirconium bead–prefilled tubes (OPS Diagnostics, Lebanon, NJ, USA). Muscle tissue was homogenized in TriPure isolation reagent (Roche Diagnostics, Basel, Switzerland), with a MagNA Lyser (Roche Diagnostics). Total RNA was isolated by the TriPure isolation method, and the RNA was further cleaned up by applying a NucleoSpin RNA II kit (Macherey-Nagel, Düren, Germany), according to the manufacturer’s instruction. cDNA was synthesized from 0.5 to 1 μg of RNA using random N6 primers (Thermo Fisher Scientific) and Bioscript enzyme (GCBiotech, Alphen aan den Rijn, The Netherlands) according to the manufacturer’s instructions. For exon skip analysis, 10 times diluted cDNA was amplified by PCR with FastStart Taq DNA Polymerase (Roche Diagnostics) followed by use of the Lab-on-a-Chip system (Agilent Technologies, Santa Clara, CA, USA), to measure exon skip levels (22). Quantitative PCR (qPCR) was performed in triplicate per biologic sample, with LightCycler 480 and the ready-to-use SensiMix reagents (GCBiotech). The expression levels were analyzed by applying the LinReg qPCR method and normalized to expression values of the housekeeping genes *Gapdh* and *Hbms*. We confirmed that their expression values did not vary between time points and animal models. Primer sequences and detailed PCR conditions will be provided on request. The exon skip levels were quantified with the Lab-on-a-Chip system (22).

### Sanger sequence analysis

RT-PCR products were isolated from 2% agarose gels with the QiaQuick Gel Extraction Kit (Qiagen). Direct DNA sequencing took place in the Leiden Genome Technology Center, with the BigDye Terminator Cycle Sequencing Ready Reaction kit. Analysis was performed on an ABI 3730 Sequencer (both from Thermo Fisher Scientific).

### RNA sequencing

Total RNA was extracted from the gastrocnemius muscles isolated from WT mice treated with ALK4 (*n =* 6) or control (*n =* 6) ViM for 7 wk. The RNA integrity number was >7.8 for all samples. RNAseq was performed at the Leiden Service XS Sequencing Center on an HiSeq 2500 sequencer (Illumina, San Diego, CA, USA) that included sample preparation, library validation and extensive data quality control and filtering. All 12 samples underwent paired-end sequencing on the same lane. The resulting raw reads were first analyzed using FastQC v0.10.1 (Brabraham Bioinformatics, Cambridge, United Kingdom; *http://www.bioinformatics.babraham.ac.uk/projects/fastqc/* ). If a read pair was found to contain any known sequencing adapters, as shown by the FastQC overrepresented sequence module, the full adapter sequences were clipped with cutadapt v1.5, setting the minimum length flag to 20. Read pairs clipped this way were synchronized with their counterparts by using a custom tool to ensure that the pairing remained correct. Reads were subjected to base quality trimming with the default options of Sickle, v. 1.33 (*https://github.com/najoshi/sickle/*). The resulting reads served as an input for alignment. The reads were aligned to the *Mus musculus* mm10 genome sequence, with GSNAP, http://www.gvst.co.uk/gsnap.htm, v. 2014-12-23, setting the −npaths flag to 1 and the −quiet-if-excessive flag to ensure unique and only unique alignments. The generated alignment file was compressed to BAM and then name-sorted using Picard 1.120 (Broad Institute, Boston, MA, USA (*https://github.com/broadinstitute/picard/*). Fragment count per gene was generated using HTSeq-count v0.6.1.p1, setting the −stranded flag to no and −order flag to name and using the GTF of the RefSeq gene annotation prepared from the table browser (University of California, Santa Cruz, Santa Cruz, CA, USA; *https://www.genome.ucsc.edu/*). Finally, a single table with fragment counts for all samples was prepared and used for differential expression analysis.

### Differential expression and pathway analyses

Fragment counts were analyzed for differential expression between ALK4- and control ViM-treated groups. Because only 2 subjects were measured in both groups, we used 2-group comparison models. The analysis was performed on 10,541 genes for which the average CPM was above 4. We used 3 R Bioconductor packages for differential expression analysis, (*i.e.*, DESeq2, limma-voom and edger), following their guidelines (http://www.bioconductor.org/help/workflows/rnaseqGene/). Genes consistently reported as differentially expressed by all 3 packages were used in further analysis. The KEGG pathway gene sets were tested for association with the group variable using the R Bioconductor global test package (23, 24). In addition to the KEGG-based pathway analysis, the OncoFinder method was used (25, 26). We also identified biologic pathways and networks through Ingenuity Pathway Analysis (IPA; Qiagen, Redwood, CA, USA). The list of differentially expressed genes was imported into IPA and based on the differentially expressed values that IPA divided genes into (*i.e.*, up- and down-regulated genes). Biologic pathways, that were most affected by the treatment were identified with the Fisher exact test (*P* < 0.05). The upstream regulator analysis algorithm identified main upstream regulators and transcriptional factors. All 3 pathway analyses were performed on counts normalized with limma-voom.

### Muscle histology and morphology

Frozen muscle sections (8 μm thickness) were cut with a cryotome and fixed in ice-cold acetone for 5 min. For measuring the fibrotic area, gastrocnemius muscle sections were stained with hematoxylin and eosin (Sigma-Aldrich) and mounted in Permount mounting medium (Histolab, Västra Frölunda, Sweden), according to conventional methods. The stained sections were examined under a light microscope (LeicaDMLB; Leica Microsystems, BV, Son, The Netherlands) at ×10 magnification. Images were taken with a Leica DC500 camera and Leica IM50 software (Leica Microsystems). The fibrotic/necrotic percentage of the entire cross section was determined with the freely available ImageJ software (NIH) (27, 28). Fiber size was measured with SMASH (Matlab based analysis; MatLab, Natick, MA, USA) (29). For these analyses, gastrocnemius muscle sections were fixed with ice-cold acetone and stained with a laminin primary antibody (ab11575, dilution 1:200; Abcam, Cambridge, MA, USA) overnight at 4°C and with a goat-anti-rabbit Alexa 594 secondary antibody (A11012, dilution 1:1000; Thermo Fisher Scientific) and DAPI for 1.5 h at room temperature. Sections were imaged with an SP5 or SP8 confocal microscope (Leica) at a ×10 magnification. At least 7–10 microscopic views were counted and analyzed, resulting in an average total number of 5000–7500 fibers per muscle. The minimum fiber area was set at 50 µm^2^ and the maximum fiber area was set at 10,000 µm^2^. The relative number of fibers in specified bins were counted and the results depicted in histograms. Percentages of regenerative areas were measured with ImageJ analysis by dividing the total embryonic myosin heavy chain (eMHC)–positive area by the total tissue area on stitched gastrocnemius muscle sections. For these analyses, gastrocnemius muscle sections were stained with an eMHC primary antibody (F1.652, dilution 1:100; Developmental Studies Hybridoma Bank, Iowa City, IA, USA) overnight at 4°C and with a goat anti-mouse Alexa 488 secondary antibody (A11001, dilution 1:1000; Thermo Fisher Scientific) and DAPI for 1.5 h at room temperature. Images of the stained muscle sections were taken with an SP5 or SP8 confocal microscope (Leica) at a ×10 magnification and stitched with the Leica software LASFx. Fiber-type switch analyses were performed with ImageJ software on a whole (stitched) gastrocnemius muscle section. Nonfixed frozen sections were stained with a laminin primary antibody (ab11575, dilution 1:200; Abcam) for 1 h at room temperature and with a donkey-anti rabbit Alexa 647 secondary antibody for 1 h at 4°C. Thereafter, slides were stained with directly conjugated fiber-type-specific antibodies: Type 1 antibody BA-D5^-Alexa 350^ (1:300), Type 2A antibody SC-71-^Alexa594^ (1/900), and Type 2B antibody BF-F3 ^–Alexa488^ (1:600) overnight at 4°C (antibodies kindly provided by Prof. D. J. Wells, Royal Veterinary College, London, United Kingdom). Conjugation was performed according to the manufacturer’s instructions with Alexa Fluor 350, 488, and 594 Protein labeling kits (Thermo Fisher Scientific). Images of the stained muscle sections were obtained with an SP5 or SP8 confocal microscope (Leica) at ×10 magnification, and images were stitched with Leica software. The percentage of fiber-type–positive areas was divided by the total muscle area.

### Statistical analyses

Data were analyzed by using Prism 4 (GraphPad Software, La Jolla, CA, USA) and SPSS 17.0.2 (IBM, Armonk, NY, USA). Values are presented as means ± sd. Data from *Alk4* and control-treated muscles were compared by Student’s *t* test. In studies that involved only a comparison between the left and right gastrocnemius or tibialis anterior muscle, a paired Student’s *t* test was used to determine statistical significance. For physiologic analysis of tibialis anterior muscles, repeated-measures ANOVA and multiple comparisons with Bonferroni were used. To access fiber size distribution, 2 different statistical tests were conducted with SPSS: logistic regression to demonstrate the switch toward smaller fiber sizes for *Alk4* ViM treatment; and mixed model for WT samples and a paired Student’s *t* test for *mdx* samples, to access significant differences in mean fiber area between control and *Alk4* ViM-treated muscles. Statistical significance was set at *P* < 0.05.

## RESULTS

### AON-mediated exon skipping of *Alk4* enhances myogenic differentiation *in vitro*

**Figure 1.**
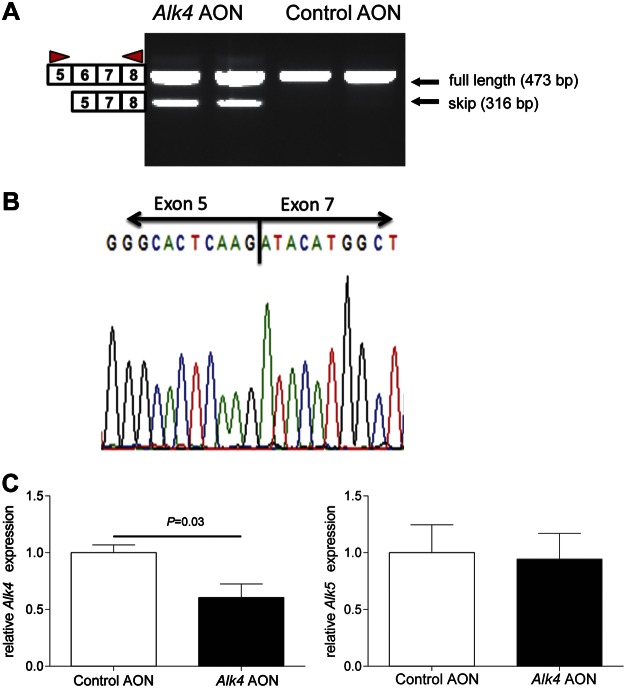
*Alk4* AON targets the out-of-frame exon 6 and results in efficient exon 6 skipping and *Alk4* knockdown in C2C12 myoblasts. *A*) AON targeting exon 6 of *Alk4* (*Alk4* AON) and scrambled control AON were transfected at 100 nM concentration into C2C12 murine myoblasts. RNA was isolated after 24 h, and the efficiency of exon-skipping induction was measured by RT-PCR using *Alk4*-specific primers in flanking exons (arrowheads). *B*) Sequencing of the PCR products showed exclusion of the target exon 6 (exon 6 skip). *C*) qPCR was performed to measure full-length *Alk4* transcript expression using primers in the skipped exon to assess *Alk4* transcript down-regulation. *Alk5* transcript expression was measured by qPCR and remained unchanged in *Alk4* AON-treated C2C12.The data are presented as the average of the results of 3 independent experiments normalized to *Gapdh* and are shown relative to control AON samples. Error bars, sd.

**Figure 2.**
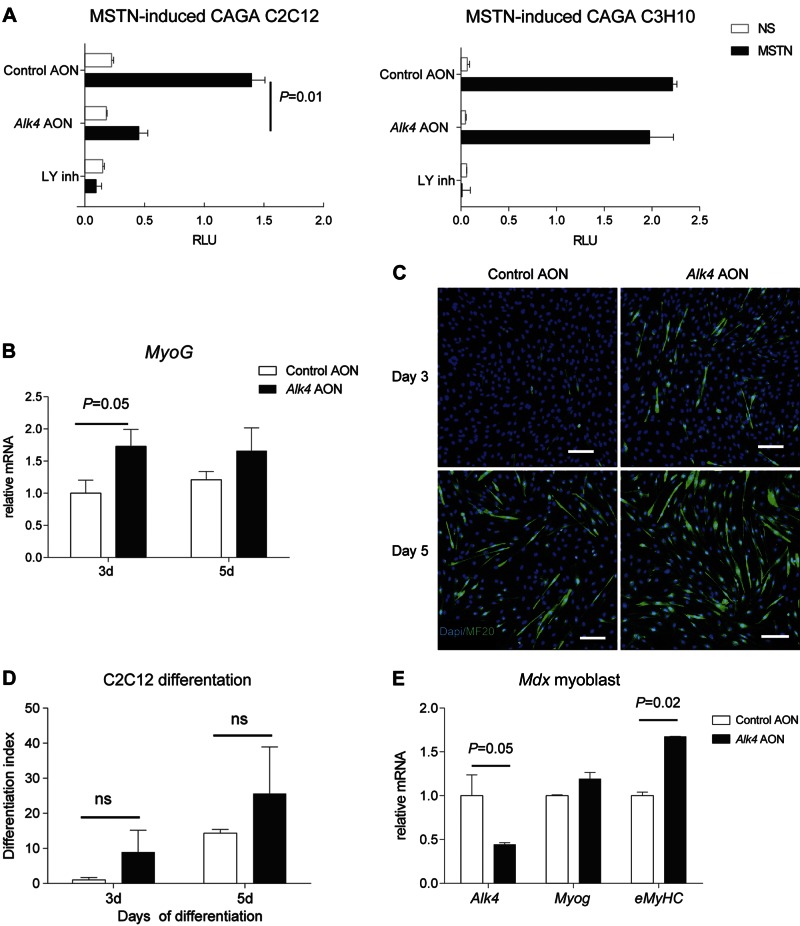
*Alk4* AON blocks MSTN signaling and enhances myoblast differentiation. *A*) *Alk4* AON (200 nM) specifically repressed MSTN-induced Smad3-dependent (CAGA)_12_-luciferase activity in C2C12 murine myoblasts, whereas *Alk4* AON did not inhibit MSTN activity in C3H10T1/2 mesenchymal stem cells. LY364947 inhibitor (LY inh; 10 μM) was added to the cells as a positive control for blockage of MSTN activity. The cells were stimulated with MSTN or nonstimulated (NS). Firefly luciferase activity of (CAGA)_12_-luciferase constructs were normalized to *Renilla* luciferase activity of cotransfected CMV-renilla constructs. The normalized values are in relative light units (RLU). *B*) qPCR was performed to measure the *Myog* expression of C2C12 cells transfected with *Alk4* or control AON (100 nM) at 3 and 5 d after initiation of myogenic differentiation. *Alk4* AON increased the *Myog* expression. *C*) Immunofluorescence images of C2C12 cells transfected with *Alk4* or control AON (100 nM) at 3 and 5 d after initiation of myogenic differentiation. Cells were stained with myosin (differentiated myotubes, green) and DAPI (nuclear, blue). Scale bars, 10 µm. *D*) The differentiation index was calculated as the percentage of the total number of myogenic cells that were myosin^+^. *E*) AON targeting exon 6 of *Alk4* (*Alk4* AON) and scrambled control AON were transfected at 50 nM concentration into *mdx* myoblasts. RNA was isolated after 48 h. Real-time qPCR was performed to measure full-length *Alk4* transcript down-regulation and myogenic gene expression. The data are presented as the average of the results of 3 independent experiments normalized to *Gapdh* and are shown relative to control AON samples. Error bars, sd.

An AON-mediated exon skipping strategy was developed to selectively block the function of the ALK4 receptor. AONs with specific sequences to target the out-of-frame exon 6 were designed by a published protocol (30), resulting in the skipping of this exon and knockdown of *Alk4* transcript. Newly designed AONs with phosphorothioate backbones and 2′-*O*-methyl ribose modifications (2OMePS) were used in our *in vitro* study. The efficiency of each AON was assessed by measuring exon-skipping levels and transcript down-regulation after transfection in C2C12 myoblasts. The most efficient AON was selected for further studies (data not shown), and a scrambled control for it was designed (henceforth referred to as *Alk4* and control AON, respectively). *Alk4* AON, but not the control AON, efficiently induced exon 6 skipping after transfection in C2C12 myoblasts as measured by RT-PCR analysis with primers flanking the target exon 6 (**Fig. 1*A***). Sequencing of the PCR skip product showed that the targeted exon 6 was excluded (Fig. 1*B*). In addition, a specific 40% decrease in *Alk4* full-length transcript was measured by real-time qPCR analysis with primer pairs both inside and outside exon 6 (Fig. 1*C*). *Alk4* AON led to a specific transcript down-regulation as *Alk5* expression did not differ between control and *Alk4* AON-transfected C2C12 cells, whereas *Alk7* was not expressed by C2C12 cells (data not shown). Next, we studied the effect of *Alk4* AON on protein level and function. Because of a lack of specific ALK4 antibodies, we looked at the downstream effects of *Alk4* AON-mediated knockdown and its ability to block MSTN signaling *in vitro*. In a previous study, we found that MSTN signaling is mediated *via* ALK4 in myogenic cells and *via* ALK5 in nonmyogenic cells using siRNAs (17). In the present study, C2C12 myoblasts and C3H10T1/2 cells (nonmyogenic cells) were cotransfected with *Alk4* AON and a (CAGA)12-luciferase transcriptional reporter construct that drives expression of a luciferase reporter gene in a Smad3-dependent manner (17, 31). *Alk4* AON blocked the MSTN signaling in C2C12 cells, but exerted no effect on the C3H10T1/2 cells (in which MSTN signals *via* ALK5). The ALK4/5/7 kinase inhibitor LY364947 served as a positive control for blocking MSTN signaling in both cell lines (**Fig. 2*A***).

We also studied the effect of *Alk4* AON-mediated exon skipping on C2C12 myoblast differentiation. C2C12 cells were transfected with *Alk4* AON and 4 h later, differentiation was induced by serum starvation. At d 3, C2C12 cells were transfected again with *Alk4* AON, and differentiation was monitored for up to 5 d. Expression of myogenin (*Myog*) was increased in *Alk4* AON-transfected C2C12 cells (Fig. 2*B*). *Alk4* AON-transfected cells also showed an increased number of MHC^+^ cells (marker for myotubes) normalized to the total number of DAPI^+^ cells (marker for nuclei) (Figs. 2*C*, *D*). Comparable down-regulation on the transcript level and an increase in myogenic gene expression was also detected in primary myoblasts isolated from *mdx* EDL muscles (Fig. 2*E*). In summary, partial blockage of ALK4 with AON-mediated exon skipping enhanced myoblast differentiation *in vitro*.

### Efficient *Alk4* exon skipping decreases muscle mass but has no effect on tibialis anterior muscle contraction physiology in *mdx* mice

**Figure 3.**
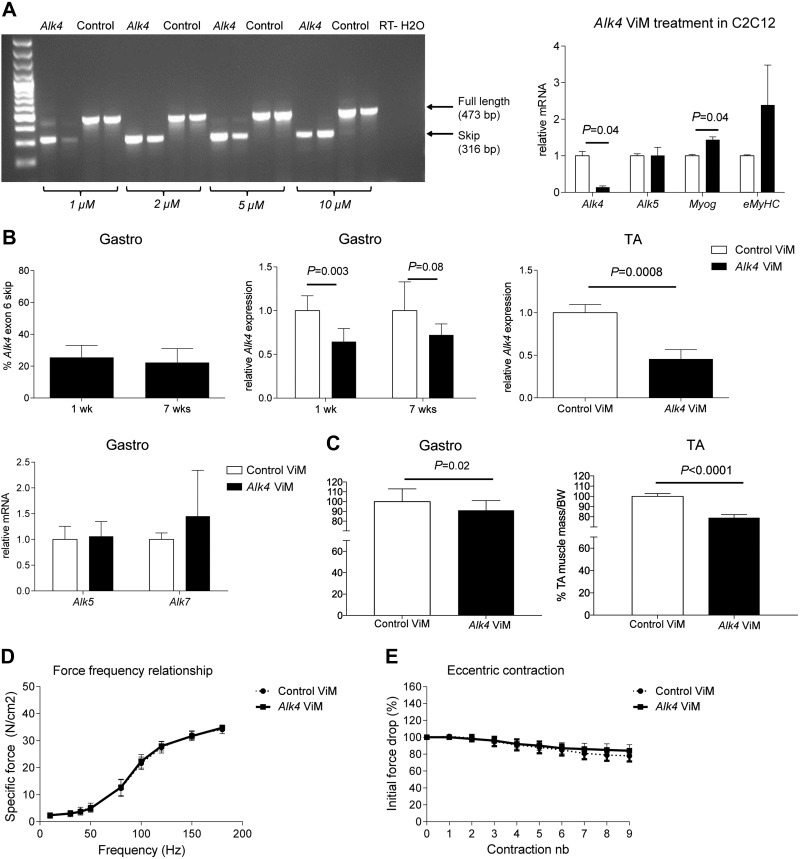
Effect of *Alk4* down-regulation with ViM in C2C12 cells, *mdx* gastrocnemius and tibialis anterior muscles. *A*) ViM targeting exon 6 of *Alk4* was added to cells without any transfection reagent at 1–10 μM for 72 h, and exon skip efficiency was measured with RT-PCR. *Alk4* and *Alk5* transcripts and myogenic gene expression were measured with qPCR of samples treated with 1 μM *Alk4* and control ViM. *B*) Percentages of exon skipping of *Alk4* or control ViM-injected gastrocnemius muscles after 1 and 7 wk of treatment were measured. qPCR was performed to measure *Alk4* full-length transcript of *Alk4* or control ViM-injected gastrocnemius and tibialis anterior muscles. Specific *Alk4* transcript down-regulation was measured in gastrocnemius muscles after 1 (*n =* 6) and 7 (*n =* 6) wk of treatment and in tibialis anterior muscles after 7 wk (*n =* 4) of treatment. qPCR was performed to measure *Alk5* and *Alk7* transcripts of long-term *Alk4* or control ViM-injected gastrocnemius muscles. No differences in either transcript were detected after the treatment. *C*) Weighing of the *Alk4* ViM-treated gastrocnemius muscles showed a 10% decrease, and tibialis anterior muscles showed a 20% decrease relative to control ViM. Muscle weights were normalized to the body weights at the endpoint of the experiment. *D*) Force *vs.* frequency for the tibialis anterior muscles treated intramuscularly with *Alk4* ViM and contralaterally with control ViM for 7 wk (*n =* 6). Each data point represents the force measured at each frequency. No significant differences in the total force were measured between control and *Alk4* ViM-treated muscles. *E*) Relative changes in tetanic force during 9 cycles of eccentric contraction in tibialis anterior muscles (*n =* 6) treated intramuscularly with *Alk4* ViM and in contralateral muscles treated with control ViM for 7 wk. The tetanic tension developed during the second cycle was taken as 100%. No significant differences in the initial force decrease between control and *Alk4* ViM-treated muscles were measured. Error bars, sd (*A*–*C*) and sem (*D*, *E*).

Next, we evaluated whether *Alk4* exon skipping has a positive effect on muscle regeneration *in vivo* in the *mdx* mouse model, using ViMs, as they are more efficient *in vivo*. We first assessed whether our *Alk4* ViM exerted effects comparable to those of the *Alk4* AON *in vitro*. In C2C12 cells, *Alk4* ViM led to an efficient, dose-dependent (1, 2, 5, and 10 μM naked uptake) exon 6 skipping and a significant 85% decrease in *Alk4* transcript levels (**Fig. 3*A***). Similar to the results obtained with *Alk4* AON, *Alk4* ViM significantly increased *Myog* gene expression and did not affect *Alk5* transcript expression in C2C12 cells. *Mdx* mice were intramuscularly injected with *Alk4* ViM or standard control ViM (nontargeting) in the gastrocnemius and/or tibialis anterior muscles on 2 consecutive days and euthanized 1 wk (short-term treatment) or 7 wk (long-term treatment) after the first injection. In the long-term treatment, a third injection was given 4 wk after the first. It appeared that 25% exon skipping and 30% down-regulation were achieved in the gastrocnemius muscles (Fig. 3*B*). Comparable *Alk4* down-regulation took place in the tibialis anterior muscles. We achieved a specific *Alk4* down-regulation as *Alk5* and *Alk7* transcripts remained unaffected in gastrocnemius muscles after long-term treatment. Unexpectedly, the gastrocnemius muscle mass had significantly decreased by 10% after long-term *Alk4* ViM treatment. Moreover, the tibialis anterior muscle mass had significantly decreased (by 20%) after long-term *Alk4* ViM treatment (Fig. 3*C*).

To investigate whether the significant decrease in muscle mass after long-term *Alk4* ViM treatment affected muscle function, we performed tibialis anterior physiology analysis with a focus on the force–frequency relationship and resistance to eccentric contractions. The force–frequency curve of fast-twitch tibialis anterior muscles stayed flat at low frequencies and rose rapidly from 50 Hz onward (Fig. 3*D*). In relation to type of treatment, we found that tibialis anterior muscles treated with *Alk4* ViM did not generate a lower specific force than in control ViM-treated muscles over a wide range of stimulation intensities (10–180 Hz). We also measured the tibialis anterior muscle’s response to eccentric contraction, which can induce injury in *mdx* muscle fibers (32). Tibialis anterior muscles were repetitively stimulated at 120 Hz and stretched to 110% of their resting length. The isometric force dropped by 10% in *mdx* mice (Fig. 3*E*). There were no significant differences in this respect between control and *Alk4* ViM-treated muscles. Taken together, our results suggest that long-term *Alk4* down-regulation exerts no effect on muscle force and contraction of individual muscle fibers.

### Induction of muscle regeneration in *mdx* muscles after long-term *Alk4* down-regulation

**Figure 4.**
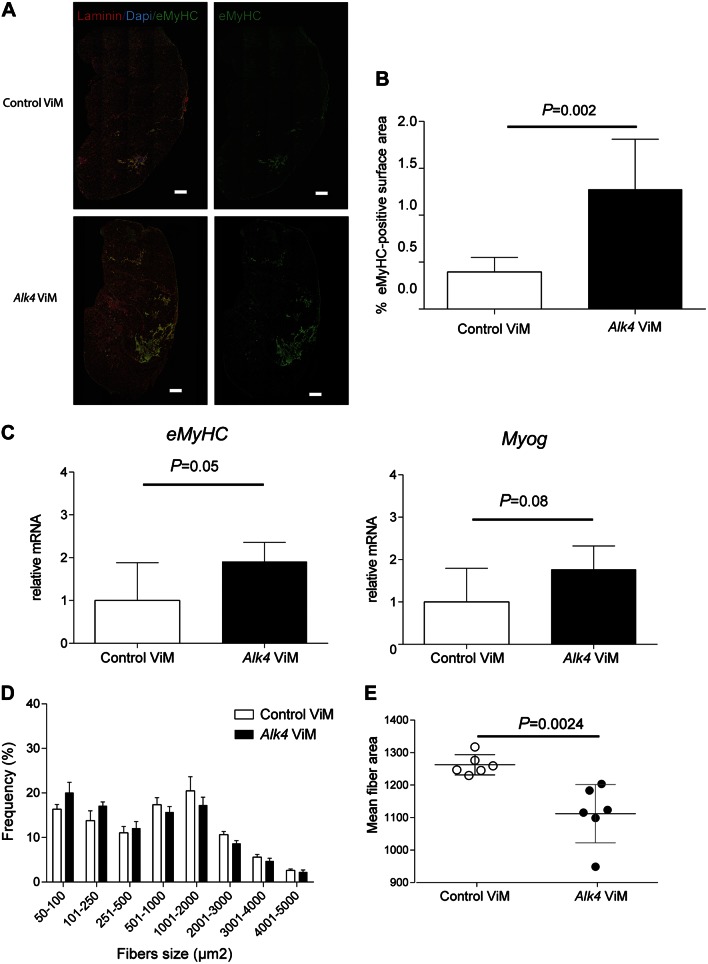
*Alk4* down-regulation increases muscle regeneration in *mdx* gastrocnemius muscles. *A*) Immunofluorescence images of *mdx* gastrocnemius muscles treated with *Alk4* and control ViM for 7 wk and stained with eMyHC (regenerative fibers, green), laminin (extracellular matrix of muscle fibers, red) and DAPI (nuclear, blue). Scale bars, 100 µm. *B*) A significant increase in eMyHC-positive area was found in muscles treated with *Alk4* ViM. *C*) Myogenic gene expression measured by qPCR, normalized to *Gapdh* (*n =* 6). Up-regulation of myogenic gene expression (*Myog* and *eMyHC*) was found after 7 wk of *Alk4* ViM intramuscular injections. *D*) The minimal fiber area was set at 50 µm^2^ and the maximum fiber area at 10,000 µm^2^. Between 7 and 10 microscopic views were counted and analyzed, resulting in an average total number of 5000–7500 fibers per muscle. *Alk4* ViM-treated muscles showed a significantly higher number of smaller fibers (50-1000 µm^2^) and a significantly lower number of larger fibers (1001–5000 µm^2^) than control ViM-treated muscles. *E*) The mean fiber area was significantly decreased after treatment with *Alk4* ViM in muscles. Error bars, sd.

In search for the mechanisms behind this unexpected lowering of the muscle mass and considering that regenerative fibers are smaller, we looked at induction of muscle de- and regeneration after *Alk4* exon skipping. The latter would be in line with the observed increase in myoblast differentiation after *in vitro Alk4* AON transfection. The effect of *Alk4* transcript down-regulation on muscle regeneration in *mdx* mice was examined by looking at *eMyHC* expression on RNA and protein level. The eMyHC^+^ area significantly increased in gastrocnemius muscle after long-term *Alk4* ViM treatment (1.5 *vs.* 0.5% surface area for *Alk4* and control ViM-treated muscles, respectively) (**Fig. 4*A*, *B***). Moreover, an increase in muscle regeneration was confirmed by qPCR analysis measuring the myogenic gene expression (*eMyHC* and *Myog*) in *Alk4* ViM gastrocnemius muscles (Fig. 4*C*). We measured the effect of *Alk4* ViM treatment on fiber size distribution as regenerative fibers are smaller in size. To demonstrate whether the switch toward smaller fiber sizes for *Alk4* ViM treatment was significant, we applied logistic regression. This analysis revealed that the number of smaller fibers (50–1000 µm^2^) in *Alk4* ViM-treated gastrocnemius muscles had significantly increased, whereas the number of larger fibers (1001–5000 µm^2^) had significantly decreased (*P* < 0.001; Fig.4*D*). The mean fiber area decreased significantly (*P* = 0.0024) after *Alk4* ViM treatment (Fig. 4*E*).

### *Alk4* exon skipping has no effect on fibrosis and fiber-type composition in *mdx* muscles

To investigate the effect of *Alk4* knockdown on muscle quality, we measured the fibrotic area in gastrocnemius muscles treated with *Alk4* or with control ViM (Supplemental Fig. S1*A*). The size of the fibrotic areas did not differ significantly between the 2 treatments, nor did the expression levels of genes involved in fibrosis: collagen type Iα1(*Col1a1*), plasminogen activator inhibitor type 1 (*Pai-1*), and connective tissue growth factor (*Ctgf*) (Supplemental Fig. S1*B*). This finding implies that *Alk4* down-regulation does not affect gene regulation for fibrotic genes in dystrophic muscles. To study whether the decrease in muscle mass after *Alk4* ViM treatment could have been the result of altered fiber-type composition, we compared the relative gene expression levels of the *MyHC* isoforms *IIx*, *IIa*, and *IIb* measured by qPCR and performed immunofluorescence staining. The relative gene expression levels of *MyHC* isoforms *IIx*, *IIa*, and *IIb* (Supplemental Fig. S1*C*) and immunofluorescence analysis of type IIa and IIb fibers (Supplemental Fig. S1*D*, *E*) did not differ significantly between muscles treated with *Alk4* and control ViM.

### Effect of *Alk4* exon skipping in adult WT mice

**Figure 5.**
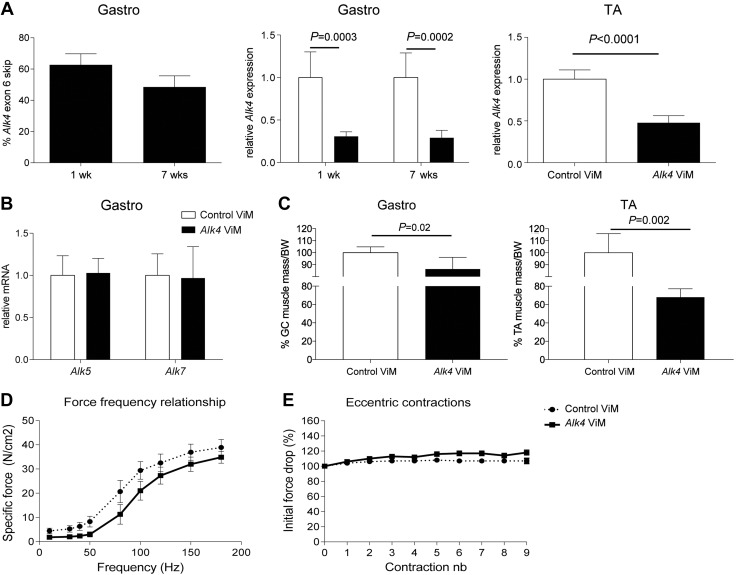
*Alk4* exon skipping decreases gastrocnemius muscle mass but has no effect on muscle physiology in WT mice. *A*) Percentages of exon skipping of *Alk4* or control ViM-injected gastrocnemius muscles after 1 and 7 wk of treatment were measured. Specific *Alk4* transcript down-regulation in gastrocnemius (*n =* 6) and tibialis anterior (*n =* 4) muscles was measured. *B*) qPCR was performed to measure *Alk5* and *Alk7* transcripts of long-term *Alk4* or control ViM-injected gastrocnemius muscles. No differences were detected after the treatment in either transcript. *C*) Weighing of the *Alk4* ViM-treated gastrocnemius muscles showed a 25% decrease and tibialis anterior muscles showed a 30% decrease in mice relative to control ViM. Muscle weights were normalized to the body weights at the endpoint of the experiment. *D*) Force *vs.* frequency for the tibialis anterior muscles of treated intramuscularly with *Alk4* ViM and contralaterally with control ViM for 7 wk (*n =* 8). Each data point represents the force measured at each frequency. No significant differences in the total force were measured between control and *Alk4* ViM-treated muscles. *E*) Relative changes in tetanic force during 9 cycles of eccentric contraction in tibialis anterior muscles (*n =* 8) treated intramuscularly with *Alk4* ViM and contralaterally with control ViM for 7 wk. The tetanic tension developed during the second cycle was taken as 100%. No significant differences in the initial force drop between control and *Alk4* ViM-treated muscles were measured. Error bars, sd (*A*–*C*) and sem (*D*, *E*).

To study whether the *Alk4*-knockdown-mediated decrease in muscle mass was developmentally regulated or induced by the dystrophic phenotype of the *mdx* mice, we injected *Alk4* ViM intramuscularly in adult WT mice and performed the same analyses as described for *mdx* mice. A significant 60% *Alk4* exon skipping and 70% transcript down-regulation was achieved in gastrocnemius muscles and comparable transcript down-regulation in tibialis anterior muscles (**Fig. 5*A***). Similar to *mdx* gastrocnemius muscles, the achieved *Alk4* down-regulation was specific, without affecting the expression of *Alk5* and *Alk7* transcripts (Fig. 5*B*). Surprisingly, we found an even more pronounced drop in muscle mass in adult WT mice (15% muscle mass drop in gastrocnemius and 30% in tibialis anterior muscles; Fig. 5*C*).

**Figure 6.**
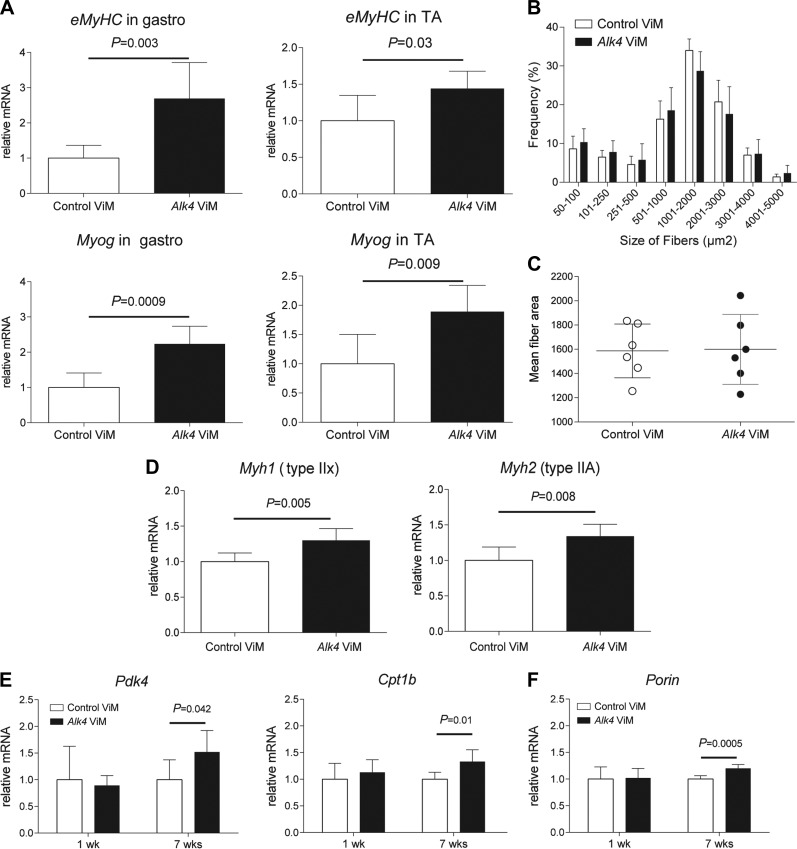
*Alk4* down-regulation induces muscle regeneration and fiber-type switch toward oxidative endurance fibers in WT gastrocnemius muscles. *A*) Myogenic gene expression was measured by qPCR. Significant up-regulation of *eMyHC* and *Myog* was found after 7 wk of *Alk4* ViM intramuscular treatment in gastrocnemius and tibialis anterior muscles. *B*) *Alk4* ViM-treated gastrocnemius showed a significantly higher number of smaller fibers (50–1000 µm^2^) and a significantly lower number of larger fibers (1001–5000 µm^2^) than control ViM-treated muscles. *C*) The mean fiber area was not significantly changed after treatment with *Alk4* ViM. *D*) A significant up-regulation of *MyHC IIx* and *IIa* isoforms was found in *Alk4* ViM-treated muscles. *E*) Gene expression of *Pdk4* and *Cpt1b* was significantly increased after 7 wk of *Alk4* ViM treatment. *F*) The gene expression of *Porin* was significantly increased at 7 wk in *Alk4* ViM-treated muscles. Gene expression levels were normalized to *Gapdh* and are depicted relative to control-treated muscles. Error bars, sd.

As with *mdx* mice, the long-term *Alk4* down-regulation and a significant muscle mass decrease did not show any effect on muscle force and contraction in adult WT mice (Fig. 5*D*, *E*). We also studied the effect of *Alk4* transcript down-regulation on muscle regeneration. A significant increase in muscle regeneration was detected by qPCR analysis measuring the myogenic gene expression (*eMyHC* and *Myog*) in *Alk4* ViM gastrocnemius muscles (**Fig. 6*A***). Comparable *eMyHC* and *Myog* gene up-regulation was found in tibialis anterior muscles. The regenerative area was not measured, because only a few eMyHC^+^ fibers were detectable by immunohistochemistry. In addition, we looked at the effect on fiber size distribution and we found that the number of smaller fibers (50–1000 µm^2^) in *Alk4* ViM-treated gastrocnemius muscles had significantly increased, whereas the number of larger fibers (1001–5000 µm^2^) had significantly decreased (*P* = 0.026) (Fig. 6*B*), but the mean fiber area remained unchanged (Fig. 6*C*).

We also measured the fibrotic area in WT gastrocnemius muscles treated with *Alk4* or with control ViM. As seen in *mdx* mice, the size of the fibrotic areas did not significantly differ between the 2 treatments, nor did the expression levels of genes involved in fibrotic gastrocnemius muscles (Supplemental Fig. S2*A*, *B*). Last, we studied the effect of *Alk4* ViM treatment on fiber type composition. In *Alk4* ViM-treated muscles the levels of the *Myh1* (*MyHC IIx*) and *Myh2* (*MyHC IIa*) isoforms had significantly increased relative to control ViM-treated muscles (Fig. 6*D*); however, no significant changes were found for *Myh7* (*MyHC I*) and *Myh4* (*MyHC IIb*) levels (data not shown). Immunohistochemistry showed a slight, nonsignificant increase in type IIa fibers and no difference in type IIb fibers in *Alk4* ViM-treated muscles (Supplemental Fig.S2*C*, *D*). This finding indicates that *Alk4* knockdown in muscles led to an increase in oxidative endurance fibers, but no reduction in fast-twitch glycolytic fibers. This result was further supported by the finding that mRNA levels of *Pdk4*, an inhibitor of pyruvate dehydrogenase and an activator of oxidative metabolism (33), was significantly up-regulated after long-term *Alk4* ViM treatment (Fig. 6*E*). We thus expected an increase in β oxidation in response to *Alk4* down-regulation and indeed found a significant up-regulation of carnitine palmitoyltransferase 1b (*Cpt1b*) mRNA levels in long-term–treated muscles. In addition, we found a significant increase in *Porin* mRNA transcript (Fig. 6*F*), which is a marker for mitochondrial function (34).

### *Alk4* down-regulation induces muscle atrophy *via* ATF4 in WT mice

To obtain an unbiased view of the genes and molecular pathways affected in response to *Alk4* down-regulation, we decided to perform RNAseq. Samples from WT gastrocnemius muscles subjected to long-term treatment with *Alk4* ViM (*n =* 6) or control ViM (*n =* 6) were sequenced using the Illumina sequencing platform, given that the drop in muscle mass was most pronounced. After cleaning and quality evaluation, each sample contained ∼20 million useful reads. The reads were aligned to the *Mus musculus* mm10 genome sequence; >95% of transcripts were mapped uniquely. Differentially expressed gene analysis was performed on 10,541 genes using 3 R Bioconductor packages: DESeq2 (35), limma-voom (36) and edgeR (37). Read counts for all samples were well normalized (Supplemental Fig. S3*A*). All 3 packages reported ∼1000 differentially expressed genes upon *Alk4* ViM treatment (Supplemental Fig. S3*B*), with *Alk4* being the most significant down-regulated gene (dot corresponding to *Alk4* indicated by an asterisk in Supplemental Fig. S3*C*). The expression analyses revealed 560 genes to be up-regulated and 310 to be down-regulated consistently in all 3 analyses (Supplemental Fig. S3*C*). To identify biologic pathways and networks affected in response to *Alk4* down-regulation, KEGG pathway gene sets were tested for associations using the R Bioconductor global test package (23) (Table 2). The metabolic pathways leading to muscle starvation and amino acid deprivation appeared to be the most affected pathways in response to *Alk4* down-regulation. In addition, the folding, sorting, and degradation pathway was activated, which is also known as unfolded protein response (UPR) or endoplasmic reticulum stress. Using the OncoFinder biomathematical method (25, 26), we were able to distinguish the activator and repressor role of every gene in each pathway of the gene expression data for individual samples (Table 3). Similar to KEGG findings, protein degradation pathways were activated by *Alk4* down-regulation, whereas protein and glucose synthesis were inhibited leading to muscle wasting. Furthermore, the cluster of differentiation (CD) 40 pathway was highly activated, which is known to induce a proinflammatory response *via* tumor necrosis factor receptor–associated factors (TRAF2 and -3) (38). Further, pathways known to induce inflammation, such as IL2 and TNF-α signaling pathways (39), were activated in response to treatment.

**TABLE 2.** Biologic pathways affected significantly in response to Alk4 down-regulation using KEGG-based analysis

Description	Class	*P*
Alanine, aspartate and glutamate metabolism	Amino acid metabolism	1.86E^–08^
Vitamin B6 metabolism	Metabolism of cofactors and vitamins	2.22E^–08^
Glycine, serine and threonine metabolism	Amino acid metabolism	1.15E^–07^
Nitrogen metabolism	Energy metabolism	1.28E^–07^
Amino sugar and nucleotide sugar metabolism	Carbohydrate metabolism	1.75E^–07^
p53 signaling pathway	Cell growth and death	6.68E^–07^
Metabolic pathways	Metabolic pathways	9.52E^–07^
One carbon pool by folate	Metabolism of cofactors and vitamins	1.10E^–06^
Lysosome	Transport and catabolism	1.31E^–06^
Aminoacyl-tRNA biosynthesis	Translation	1.33E^–06^
NOD-like receptor signaling pathway	Immune system	1.85E^–06^
α-Linolenic acid metabolism	Lipid metabolism	2.02E^–06^
Starch and sucrose metabolism	Carbohydrate metabolism	2.14E^–06^
Long-term depression	Nervous system	2.68E^–06^
RNA transport	Translation	3.04E^–06^
Glycerophospholipid metabolism	Lipid metabolism	6.04E^–06^
Arginine and proline metabolism	Amino acid metabolism	8.57E^−06^
Biosynthesis of unsaturated fatty acids	Lipid metabolism	8.73E^–06^
Protein processing in endoplasmic reticulum	Folding, sorting and degradation	8.95E^–06^
Glycosaminoglycan biosynthesis-heparan sulfate	Glycan biosynthesis and metabolism	9.04E^–06^
Valine, leucine and isoleucine biosynthesis	Amino acid metabolism	9.66E^–06^
TGF-β signaling pathway	Signal transduction	9.97E^–06^
Fat digestion and absorption	Digestive system	1.51E^–05^
Phagosome	Transport and catabolism	1.66E^–05^
Bile secretion	Digestive system	1.92E^–05^
Gap junction	Cellular community	2.08E^–05^
Proximal tubule bicarbonate reclamation	Excretory system	2.11E^–05^
Carbohydrate digestion and absorption	Digestive system	2.57E^–05^
Histidine metabolism	Amino acid metabolism	2.64E^–05^
Alzheimer's disease	Neurodegenerative diseases	2.66E^–05^
Gastric acid secretion	Digestive system	2.70E^–05^
Amyotrophic lateral sclerosis (ALS)	Neurodegenerative diseases	3.53E^–05^
Protein export	Folding, sorting and degradation	4.04E^–05^
ErbB signaling pathway	Signal transduction	4.13E^–05^
mTOR signaling pathway	Signal transduction	4.54E^–05^
Glutathione metabolism	Metabolism of other amino acids	5.24E^–05^
Aldosterone-regulated sodium reabsorption	Excretory system	6.55E^–05^
Cell cycle	Cell growth and death	8.52E^–05^
Oxidative phosphorylation	Energy metabolism	8.62E^–05^
Basal transcription factors	Transcription	1.01E^–04^
RNA degradation	Folding, sorting and degradation	1.15E^–04^
Glycolysis/gluconeogenesis	Carbohydrate metabolism	1.16E^–04^
Proteasome	Folding, sorting and degradation	1.48E^–04^
Adipocytokine signaling pathway	Endocrine system	1.49E^–04^
Pancreatic secretion	Digestive system	1.59E^–04^
Hepatitis C	Infectious diseases	1.67E^–04^
Acute myeloid leukemia	Cancers	1.99E^–04^
Parkinson's disease	Neurodegenerative diseases	2.13E^–04^

NOD, nucleotide-binding and oligomerization domain, leucine-rich repeat.

**TABLE 3.** Signaling pathways activated/inhibited significantly in response to Alk4 down-regulation using the OncoFinder method

Name	State	*P*
GSK3 pathway (gene expression *via* CTNNB1)	Activated	9.34E^–07^
GSK3 pathway (b-CTNN degradation)	Activated	1.02E^–05^
JAK mStat Pathway (JAK degradation)	Inhibited	1.02E^–05^
Ubiquitin proteasome–dependent proteolysis pathway	Activated	4.00E^–05^
WNT pathway (cell fate proliferation, differentiation, adhesion and survival)	Unclear	4.77E^–05^
CD40 pathway	Activated	2.17E^–04^
TNF main pathway	Activated	2.51E^–04^
p38 pathway (translation)	Unclear	3.27E^–04^
IL-2 pathway (IL-2 gene expression)	Activated	5.14E^–04^
SMAD signaling network pathway	Unclear	7.27E^–04^
GSK3 pathway (glycogen synthesis)	Inhibited	1.04E^–03^
mTOR main pathway	Activated	1.42E^–03^
WNT main pathway	Unclear	1.47E^–03^
PTEN pathway (apoptosis)	Inhibited	2.00E^–03^
GSK3 pathway (protein synthesis)	Inhibited	2.41E^–03^
TNF pathway (gene expression and cell survival)	Activated	2.41E^–03^
AKT main pathway	Activated	2.48E^–03^
IL-2 main pathway	Activated	2.88E^–03^
IGF1R pathway (glycogen synthesis)	Inhibited	3.91E^–03^

GSK3, glycogen synthase kinase 3; IGF1R, insulin growth factor- 1 receptor; mTOR, mammalian target of rapamycin; PTEN, phosphatase and tensin homolog; SMAD, mothers against decapentaplegic homolog 3 Wnt, wingless-type MMTV integration site family.

**Figure 7.**
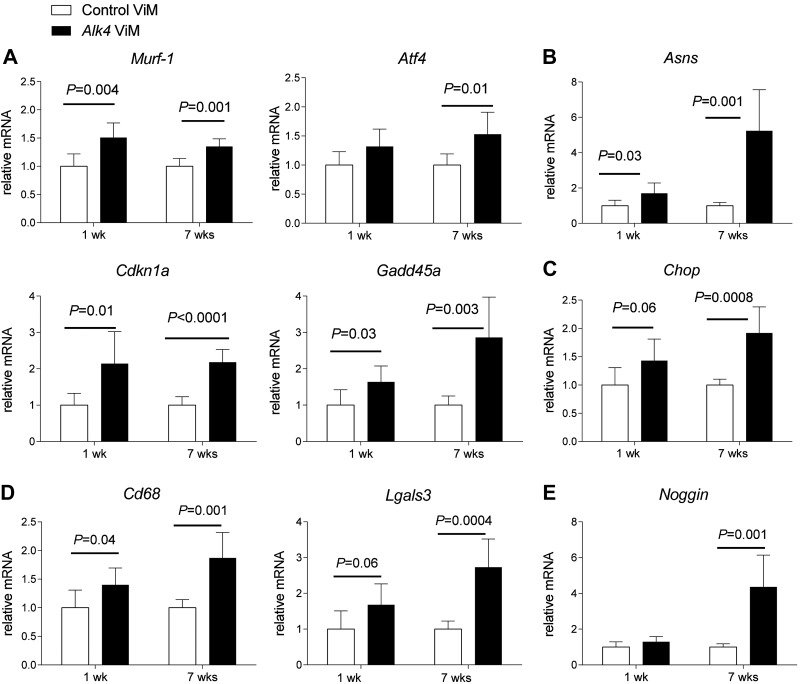
qPCR validation of WT gastrocnemius muscles after *Alk4* down-regulation. *A*) Gene expression of *Murf-1*, *Atf4*, *Cdkn1a*, and *Gadd45a* was measured with qPCR in WT gastrocnemius muscles. Significant up-regulation of *Murf-1*, *Cdkn1a*, and *Gadd45a* was found in *Alk4* ViM-treated muscles at 1 and 7 wk. Significant up-regulation of *Atf4* was found in treated muscles at 7 wk. *B*) The gene expression of *Asns* was up-regulated in muscles treated both 1 and 7 wk. *C*) The gene expression of *Chop* was up-regulated in treated muscles at both 1 and 7 wk. *D*) Significant up-regulation of *Cd68* was found at 1 and 7 wk in *Alk4* ViM-treated muscles. Significant up-regulation of *Lgals* was found at 7 wk in treated muscles. *E*) The expression of *Noggin* was up-regulated at 7 wk in *Alk4* ViM-treated muscles. All expression data were normalized to *Gapdh*. Error bars, sd.

The RNAseq analysis revealed that pathways involved in protein degradation were activated in response to *Alk4* down-regulation in WT muscles. One of the key regulators of protein degradation is muscle RING-finger protein-1 (MuRF-1/Trim63), which activates protein degradation *via* the ubiquitin proteasome (40, 41). *Murf-1* was highly up-regulated in response to *Alk4* down-regulation in our RNAseq data. We validated differences in *Murf-1* gene expression with qPCR. *Murf-1* gene expression was significantly up-regulated in response to *Alk4* down-regulation (**Fig. 7*A***).

With the Upstream Regulator Analysis algorithm of IPA, we identified activating transcription factor-4 (*Atf4*) as 1 of the most significant upstream targets (*P* = 9.13E^−26^). *Atf4* is a regulator of genes involved in muscle atrophy, such as growth arrest and DNA-damage inducible α (*Gadd45a*), cyclin-dependent kinase inhibitor 1a (*Cdkn1a*), and ankyrin repeat domain 1 (*Ankrd1*) (42, 43). *Atf4*, *Cdkn1a*, *Gadd45a*, and *Ankrd1* genes were significantly up-regulated in the RNA-seq data after long-term treatment with *Alk4* ViM. qPCR analysis confirmed a significant increase in *Atf4*, *Cdkn1a*, and *Gadd45a* in these samples (Fig. 7*A*). This effect was also detected after 1 wk of treatment, suggesting a very fast activation of the atrophic response.

The KEGG and OncoFinder-based pathway analyses showed that the most affected pathways in response to *Alk4* down-regulation were metabolic pathways resulting from muscle starvation and amino acid deprivation. One of the markers of low glucose level, asparagine synthetase (*Asns*), was highly up-regulated in response to *Alk4* down-regulation. A significant increase in *Asns* gene expression was confirmed by qPCR analysis in gastrocnemius muscles after 1 and 7 wk of *Alk4* ViM treatment (Fig. 7*B*). In addition to the metabolic changes, both KEGG-based pathway analysis and IPA showed a significant up-regulation of UPR after *Alk4* down-regulation. A significant increase in DNA damage inducible transcript 3 (*Ddit3*, or *Chop*) gene expression (a marker of UPR that is activated *via Atf4*) after 7 wk of *Alk4* ViM treatment was confirmed with qPCR analysis (Fig. 7*C*).

The OncoFinder method revealed that the CD40, TNF-α, and IL2 signaling pathways were activated in response to *Alk4* down-regulation. These pathways activate a proinflammatory response and thereby increase the expression of inflammatory cytokines (38, 39). We measured the expression of CD68 (*Cd68*) and lectin galactosidase-binding soluble 3 (*Lgals3*) (inflammatory markers) with qPCR and found a significant increase in *Alk4* ViM-treated muscles (Fig. 7*D*).

Using our RNAseq data, we studied the TGF-β superfamily and its signaling network. We found that *Noggin* was significantly up-regulated in response to *Alk4* down-regulation. This finding was confirmed with qPCR in *Alk4* ViM-treated muscles (Fig. 7*E*). As an antagonist of bone morphogenetic protein (BMP) signaling, Noggin prevents binding of BMP ligands to their receptors, thereby blocking the activation of BMP signaling (44). BMP signaling is crucial in maintaining muscle mass, preventing muscle atrophy, and regulating muscle regeneration (45–47). In addition, our attention was drawn to the *Fbxo30* gene, which encodes the F-box protein belonging to the F-box-containing complex family of ubiquitin. *Fbxo30* was significantly up-regulated in response to *Alk4* down-regulation (data not shown), and it has been shown to be induced in the atrophying muscles of *Smad4*-deficient mice (45).

We also assessed expression of genes involved in atrophic response in *mdx* gastrocnemius muscles treated with *Alk4* or control ViM. In contrast to the finding in WT mice, no significant differences in the atrophic markers *Cdkn1a*, *Murf-1*, and *Atf4* were found in *Alk4* ViM-treated *mdx* gastrocnemius muscles, whereas *Gadd45a* was up-regulated after 7 wk of *Alk4* ViM treatment (Supplemental Fig.S4*A*). Furthermore, a significant increase in *Asns* gene expression (low glucose marker) was found in *mdx* muscles after 1 and 7 wk of *Alk4* ViM treatment (Supplemental Fig.S4*B*). In addition to metabolic changes, a significant up-regulation of UPR after *Alk4* down-regulation was demonstrated. A significant increase in *Chop* gene expression after 7 wk of *Alk4* ViM treatment was found with qPCR analysis (Supplemental Fig.S4*B*). Last, Noggin was also increased in *mdx* muscles after 7 wk of *Alk4* ViM treatment (Supplemental Fig.S4*D*). These findings suggest that, as in WT muscle, the UPR pathway is activated by *Alk4* down-regulation, whereas atrophic pathways are not activated in *mdx* muscles.

## DISCUSSION

Many research groups have focused on blockage of the MSTN/ACVR2B pathway as a therapy for various types of muscle dystrophies (48). Progress has been made, but it is still being debated whether this blockage can improve muscle function and muscle quality (12, 49–51). MSTN signaling not only negatively regulates muscle growth, but also affects metabolic properties of the muscle, such as oxidative capacity, glucose metabolism, and fat utilization (52). Recently, it has been found that blockage of ACVR2B induces hypertrophy, but is accompanied by increased muscle metabolic myopathy (53).

In the current study, we developed a novel approach to specifically block MSTN signaling, involving targeting the ALK4 receptor with AONs, which is necessary to activate Smad transcription factors. To inhibit ALK4, we developed 2OMePS AONs and ViMs, which specifically target out-of-frame exon 6, generating a premature stop codon in exon 7. A premature stop codon can activate nonsense-mediated decay, leading to *Alk4* transcript degradation. However, we cannot rule out that skipping of exon 6, which encodes a crucial part of the kinase domain, would generate a dysfunctional ALK4 protein–lacking kinase domain that would act as a ligand trap.

The sequence-specific approach using AONs has the advantage that it specifically modulates *Alk4* expression and does not induce any off-target effects. To our knowledge, there are no other specific ALK4 inhibitors available, and most small molecules inhibit nonrelated protein kinases and related protein kinases, such as ALK5 and ALK7 (54). Indeed, with our approach we showed specific knockdown of *Alk4* expression after transfection or injection with *Alk4* AONs in cells and in mice. Because of the lack of specific ALK4 antibodies, we were not able to show the effect on protein level. However, the specific inhibition of MSTN signaling and the enhanced myogenic differentiation shown after *Alk4* AON transfection in myogenic cells suggest that we achieved functional inhibition of MSTN signaling with *Alk4* AON *in vitro*.

Unexpectedly, *Alk4* down-regulation *in vivo* resulted in a profound, significant decrease in muscle weight. This result contradicts findings from many other groups—that is, an increase in muscle mass in response to MSTN/ACVR2B blockage (12, 13, 49, 50, 55). However, Smad3 depletion decreases muscle mass as well and triggers pronounced muscle atrophy (56, 57). Furthermore, Smad3 is essential for normal skeletal muscle growth and muscle regeneration, and depletion of Smad3 increases *Mstn* expression, thus contributing to muscle atrophy and impaired muscle regeneration. In our *Alk4* down-regulation model, we did not detect any increase in *Mstn* expression (data not shown) suggesting that muscle atrophy is triggered *via* different mechanism.

### The role of ALK4 in muscle regeneration

To identify the cause of *Alk4*-knockdown–mediated muscle weight decrease, we first looked at the influence of muscle degeneration/regeneration, because regenerative fibers are smaller and increased regeneration may partially explain the observed drop in muscle weight. The rationale for this approach was the observed increased differentiation after *Alk4* knockdown *in vitro*. The *mdx* mice showed increased regenerative areas upon *Alk4* AON treatment and a significant overall decrease in fiber size by ∼150 μm^2^. This effect was not found in WT mice, which however, had a more significant lowering in muscle mass. It should be mentioned that, the regenerative effect in *mdx* mice was only minor (1.5% increase, compared to a 10% decrease in muscle mass). Thus, increased regeneration does not seem to be the primary cause of the decrease in muscle mass. Seeing that MSTN can affect the metabolic profile of skeletal muscles (52), another possibility thus would be a shift from larger type IIx to smaller type IIa fibers. We found that *Alk4* down-regulation led to higher mRNA levels of markers for oxidative endurance fibers and markers for a shift toward an oxidative metabolism and away from glycolytic metabolism. This finding implies that ALK4 controls the metabolic profile of skeletal muscle and that blockage of ALK4 improves oxidative properties, thereby possibly improving the endurance capacity of skeletal muscles. Notably, blockage of the other MSTN coreceptor ACVR2B had an opposite effect on the metabolic profile [*i.e.*, a shift toward a glucose metabolism, increased muscle fatigability, and mitochondrial dysfunction (53)]. Further work is needed to elucidate the metabolic function of MSTN and its type I and II receptors in healthy and dystrophic models.

### The role of ALK4 in muscle atrophy

RNAseq analysis showed increased gene expression of the key regulators of muscle degradation (*Murf-1*) and muscle atrophy (*Atf4*). MuRF-1 is one of the key activators of the ubiquitin proteasome pathway, leading to protein breakdown (40). *Murf-1* mRNA is therefore a good biomarker for proteolysis and rapid muscle wasting. Activating transcription factor *(*ATF4) is tightly regulated and activates many genes involved in the cellular response to stress (58). For instance, prolonged food withdrawal increases *Atf4* expression, which mediates myofiber atrophy. Furthermore, *Atf4*-mediated transcriptional regulation is essential for myofiber atrophy (43). *Atf4* expression increases the expression of a subset of genes involved in starvation-induced myofiber atrophy. In the present study, this subset of genes—*Gadd45a*, *Cdkn1a*, and *Ankrd1*, together with *Atf4* and *Murf-1*—were up-regulated in *Alk4* ViM-treated WT muscle, as evidenced by the RNAseq data and confirmed by qPCR. Taken together, these data suggest that *Alk4* down-regulation activates protein degradation *via* the ubiquitin proteasome pathway and induces starvation-induced muscle atrophy *via* ATF4 signaling. We confirmed up-regulation of genes involved in myofiber atrophy in WT gastrocnemius muscles upon *Alk4* down-regulation. Up-regulation of *Murf-1* and atrophic genes (*Gadd45a*, *Cdkn1a*) was observed as early as 1 wk after *Alk4* AON treatment, suggesting a very rapid atrophic response to *Alk4* down-regulation. In *mdx* samples, no such effect was found, but an increase in *Gadd45a* expression was measured after 7 wk of treatment. The discrepancy in atrophic response between WT and *mdx* mice can be explained by difference in age (3 mo for WT *vs.* 4 wk for *mdx*), in levels of *Alk4* down-regulation (70% for WT *vs.* 30% for *mdx*) and by the presence of the dystrophic pathology in *mdx* mice, which triggers activation of inflammatory, fibrotic, and degenerative/regenerative pathways.

### The role of ALK4 in muscle metabolism and UPR activation

Our data suggest that decreased ALK4 receptor levels in muscles lead to altered metabolism and low levels of glucose homeostasis, causing amino acid deprivation and muscle starvation. Because of muscle starvation, UPR is activated to recycle proteins, ultimately leading to increased atrophic response and muscle mass loss.

### *Noggin* up-regulation contributes to muscle wasting and regeneration

Recent findings of a positive role for BMP-mediated signaling in muscle challenged the idea of how the TGF-β superfamily and its signaling network regulates the skeletal muscle phenotype (59). Blockage of BMP signaling causes muscle atrophy, reduces muscle mass in MSTN-knockout mice (BMP has a dominant role over MSTN) and strongly exacerbates the effects of denervation and food withdrawal (45). In the latter study, muscle atrophy was induced by overexpression of *Noggin* (BMP antagonist). In our *Alk4* down-regulation model, we found that *Noggin* was significantly up-regulated, and the change resulted in inhibition of BMP signaling. *Noggin* up-regulation and increase in *Fbxo30* mRNA levels may explain the unexpected decrease in muscle mass. We postulate that inhibition of BMP signaling is associated with activation of protein breakdown and the starvation-induced muscle atrophy that we observed in our *Alk4* down-regulation model. BMP signaling is also known to regulate muscle regeneration. Overexpression of the BMP antagonist *Noggin* increases muscle regeneration and improves muscle quality in *mdx* mice (60). In contrast, BMP was found to be crucial for normal muscle repair after induced muscle injury in a previous study (61). In our *Alk4* down-regulation model, muscle regeneration was increased, though with a smaller fiber profile, in *mdx* mice. We postulate that this increase is linked to the inhibition of BMP signaling *via Noggin* up-regulation. However, more studies on downstream targets of BMP signaling are needed to show the link between *Alk4* down-regulation and BMP blockage.

As activins function *via* the ALK4 receptor in muscles, the effects we observed may be caused by interference with activin signaling. Activins are mediators of both inflammatory and anti-inflammatory activities and initiators of the cytokine cascades that activate an inflammatory response (62). In our *Alk4* down-regulation model, we observed *Cd68* and *Lgals3* up-regulation in WT muscles, which suggests that ALK4 not only affects myofibers but also activates proinflammatory cells such as macrophages (CD68+). In *mdx* mice, *Alk4* down-regulation did not increase gene expression of the inflammatory markers any further, probably because of an already present chronic inflammation caused by dystrophin deficiency. As previously described, *Alk4* mRNA is abundant in endothelial cells, and inhibition of MSTN or treatment with ActRIIB inhibitors negatively affects muscle capillarization (53). We studied the effect of *Alk4* down-regulation on VEGF signaling, but did not find any differences in *Vegf-A* and *Cd31* gene expression between *Alk4* ViM- and control ViM-treated muscles (data not shown). This result suggests that blockage of the ALK4 receptor does not affect capillary formation and function and that muscle starvation and atrophy do not occur because of decreased capillary density and function.

In our study, *Alk4* knockdown in *mdx* mice did not result in overt improvements of the dystrophic phenotype and muscle function. We detected only a small increase in regeneration and in the number of smaller fibers. However, after ALK4 blockage we observed an altered muscle metabolism, leading to metabolic myopathy accompanied by stress responses and amino acid deprivation. It would be worthwhile to study whether these pathways are also induced with MSTN antibodies and ACVR2B knockdown.

In summary, in this study, we showed that one of the key receptors of the MSTN pathway, the ALK4, influences myogenesis and regulates the tight balance between protein synthesis and degradation to maintain muscle mass. Therefore, caution should be used in developing therapies that involve interference in the MSTN/activin pathway.

## AUTHOR CONTRIBUTIONS

S. Pasteuning-Vuhman performed research and implemented suggestions; J. W. Boertje-van der Meulen performed *in vitro* and *in vivo* experiments; M. van Putten performed *in vitro* TA physiology and wrote related paragraphs; M. Overzier performed intramuscular injections and muscle isolations; P. ten Dijke and W. M. Hoogaars designed the research; S. M. Kiełbasa and W. Arindrarto analyzed RNA-seq data; R. Wolterbeek performed statistical analyses for fiber size distribution; K. V. Lezhnina performed pathway analyses using the OncoFinder method; I. V. Ozerov and A. M. Aliper developed the OncoFinder method; A. Aartsma-Rus designed the research and coordinated the paper-writing process; C. J. M. Loomans performed and helped design the research. S. Pasteuning-Vuhman wrote the first draft of the paper, and all remaining authors edited the paper.

## Abbreviations

2OMePS: 2'-*O*-methyl phosphorothioate
*Acvr2b*: activin A receptor type 2B
ALK: activin A receptor kinase
ALK5: transforming growth factor β receptor 1
ALK7: activin A receptor, type IC
*Ankrd*: ankyrin repeat domain
AON: antisense oligonucleotide
*Asns*: asparagine synthetase
*Atf*: activating transcription factor
*Bmp*: bone morphogenetic protein
CD: cluster of differentiation
*Cdkn*: cyclin-dependent kinase inhibitor
*Chop*: DNA damage-inducible transcript 3
*Col1a1*: collagen type Iα1
*Cpt1b*: carnitine palmitoyltransferase 1b
DMD: Duchenne muscular dystrophy
EDL: extensor digitorum longus
*eMyHC*: embryonic myosin heavy chain
FBS: fetal bovine serum
*Fbxo*: F-box protein
*Gadd*: growth arrest and DNA damage-inducible
*Gapdh*: glyceraldehyde-3-phosphate dehydrogenase
IPA: Ingenuity Pathway Analysis
*Lgals3*: lectin galactosidase-binding soluble 3
*mdx*: mouse model for Duchenne muscular dystrophy
MSTN: myostatin
*Murf*: muscle RING-finger protein
*Myh*: myosin heavy chain
*Myh4*: myosin heavy chain 4
*Myog*: myogenin
*Pdk*: pyruvate dehydrogenase kinase
qPCR: quantitative PCR
SC: serum-containing
UPR: unfolded protein response
ViM: vivomorpholino
WT: wild-type
